# The Diurnal Path to Persistent Convective Self‐Aggregation

**DOI:** 10.1029/2021MS002923

**Published:** 2022-05-23

**Authors:** Gorm G. Jensen, Romain Fiévet, Jan O. Haerter

**Affiliations:** ^1^ Niels Bohr Institute Copenhagen University Copenhagen Denmark; ^2^ Complexity and Climate Leibniz Centre for Tropical Marine Research Bremen Germany; ^3^ Physics and Earth Sciences Jacobs University Bremen Bremen Germany

**Keywords:** convection, self‐aggregation, diurnal, mesoscale, clouds

## Abstract

Clustering of tropical thunderstorms constitutes an important climate feedback because it influences the radiative balance. Convective self‐aggregation (CSA) is a profound modeling paradigm for explaining the clustering of tropical oceanic thunderstorms. However, CSA is hampered in the realistic limit of fine model resolution when cold pools—dense air masses beneath thunderstorm clouds—are well‐resolved. Studies on CSA usually assume the surface temperature to be constant, despite realistic surface temperatures varying significantly between night and day. Here we mimic the diurnal cycle in cloud‐resolving numerical experiments by prescribing a surface temperature oscillation. Our simulations show that the diurnal cycle enables CSA at fine resolutions, and that the process is even accelerated by finer resolutions. We attribute these findings to vigorous combined cold pools emerging in symbiosis with mesoscale convective systems. Such cold pools suppress buoyancy in extended regions (∼100 km) and enable the formation of persistent dry patches. Our findings help clarify how the tropical cloud field forms sustained clusters under the diurnal forcing and may have implications for the origin of extreme thunderstorm rainfall and tropical cyclones.

## Key Points

- Diurnal surface temperature oscillations can enable convective self‐aggregation in idealized simulations―especially at fine grid resolution
- Mechanistically, strong mesoscale cold pools dry the boundary layer, which later induces irreversible dry patches in the free troposphere
- Once formed, such dry patches can persist and intensify even if the diurnal cycle is later removed

## 1 Introduction

Convective self‐aggregation (CSA) refers to the spatial separation into deep convective and dry subregions occurring spontaneously in numerical simulations with homogeneous boundary and initial conditions (Bretherton et al., 2005; Held et al., 1993; Muller et al., 2022; Tompkins & Craig, 1998; Wing et al., 2017). CSA serves as a plausible mechanism for observed large‐scale tropical convective clustering, including the Madden‐Julian oscillation (Zhang, 2005) or the formation of tropical cyclones (Emanuel, 2018; Muller & Romps, 2018). Modeling suggests that CSA typically hinges on local radiation feedbacks (Coppin & Bony, 2015; Emanuel et al., 2014; Hohenegger & Stevens, 2016; Muller & Bony, 2015; Muller & Held, 2012). Maintenance of CSA has been attributed to a large‐scale circulation resulting in an upgradient moisture transport (Craig & Mack, 2013; Emanuel et al., 2014; Holloway & Woolnough, 2016; Muller & Bony, 2015). The circulation is driven by a combination of moist adiabatic lifting in the convectively active region and enhanced radiative cooling in the dry region, which must be compensated by subsidence heating.

The initial harbinger of CSA is the formation of several small persistent dry patches (Wing et al., 2017). At this initial stage, low cloud (Muller & Held, 2012) and moisture feedbacks (Emanuel et al., 2014; Muller & Bony, 2015) within dry regions were found to be critical for overcoming the re‐distribution of moisture by negative feedbacks (Bretherton et al., 2005). Cold pools (CPs)—density currents produced by rain re‐evaporation beneath thunderstorm clouds—were reported to act against such clustering (Boye Nissen & Haerter, 2019; Jeevanjee & Romps, 2013; Muller & Bony, 2015). Also finer horizontal grid resolution, which intensifies CP effects (Hirt et al., 2020; Moseley et al., 2020; Muller & Held, 2012; Yanase et al., 2020), hampered the onset of CSA.

CSA is typically studied in the context of radiative–convective equilibrium (RCE) with constant boundary conditions in both time and space (Wing et al., 2017). The RCE framework is arguably a reasonable approximation of low‐latitude oceanic conditions because sea surface temperatures exhibit only small diurnal fluctuations, especially under windy conditions (Johnson et al., 1999; Weller & Anderson, 1996). However, it is observationally evident that temporal surface temperature variations influence the spatial characteristics of convective rainfall (Bellenger & Duvel, 2009; Bellenger et al., 2010; Chen & Houze, 1997; Dai, 2001; Kawai & Wada, 2007; Peatman et al., 2014; Suzuki, 2009). For example, over tropical rain forests, where surface temperature ranges are on the order of 10 K between day and night (Sharifnezhadazizi et al., 2019), a large fraction of extreme rainfall results from mesoscale convective systems (MCSs) (Schumacher & Rasmussen, 2020; Tan et al., 2015). MCSs are defined as thunderstorm clusters exceeding 100 km spatially and 3 hours temporally (Houze, 2004). Despite indications that MCS rainfall rates and volumes might be increasing (Fowler et al., 2021; Prein et al., 2017; Westra et al., 2014), the forecast performance for MCS remains low (Fritsch & Carbone, 2004; Sukovich et al., 2014).

Several studies mimic diurnal variation through oscillating surface temperatures (Cronin et al., 2015; Liu & Moncrieff, 1998; Ruppert & Hohenegger, 2018; Ruppert & O’Neill, 2019; Tian et al., 2006; Yanase & Takemi, 2018). Under such conditions, recent simulations demonstrated the spontaneous formation of MCS‐like clusters (Haerter et al., 2020), which appeared only when the surface temperature amplitude was sufficiently large (≳3.5 K). The clusters were attributed to vigorous “combined cold pools,” which were able to force moist boundary layer air to the level of free convection. When the amplitude was smaller (≲2 K), neither MCSs nor combined cold pools were detected, and the organizational pattern was similar to the near‐random pattern during the early stages of RCE simulations. The organizational pattern observed in the presence of a large diurnal amplitude—referred to as “diurnal self‐aggregation” (DSA)—is similar to CSA in that clustering occurs spontaneously and is concentrated in parts of the spatial domain. However, DSA differs from CSA as clusters organize into patterns that are anti‐correlated from day to day, such that an area receiving pronounced rain on a given day will typically receive weak rain, or none at all, on the following. Haerter et al. (2020) showed that strong diurnal surface temperature oscillations concentrate convection and precipitation to occur predominantly during afternoon and evening, whereas the remainder of the day shows quiescent conditions. Despite very weak wind speed during quiescent periods, the atmosphere must keep some memory in order for the convective activity to have a negative correlation from day to day. Yet, it remains to be described where and how the atmosphere stores this memory.

The current study explores the causes of the memory effect: We carry out a set of idealized numerical simulations with configuration resembling those of Haerter et al. (2020). We consider a range of horizontal spatial resolutions from 0.5 to 4 km and both constant and diurnally‐oscillating surface boundary conditions. As in Haerter et al. (2020), an alternating DSA dynamics is uncovered, with strong negative correlation of moisture near the top of the boundary layer. To our initial surprise, the analysis also reveals a strong positive moisture correlation in the free troposphere. The positive correlation is associated with the emergence of persistent dry patches closely resembling the early stage of classical CSA. A constant surface temperature control experiment shows no signs of aggregation. Our findings thus identify the diurnal cycle as a causal mechanism for this persistence.

Our study reaches three main conclusions: (a) Fine resolution accelerates the emergence of persistent dry patches when the surface temperature oscillates. This identifies a key difference to classical RCE setups, where it is known that fine model resolution can hamper CSA (Jeevanjee & Romps, 2013; Muller & Bony, 2015; Muller & Held, 2012), whereas coarse resolution can support it and even mask the diurnal cycle (Yanase & Takemi, 2018). (b) For oscillating surface temperatures high resolution gives rise to mesoscale cold pools that are larger and travel faster. We ascribe this to stronger convective triggering effects under the finer resolution. For constant surface temperatures there is no such resolution effect. (c) Dry patches exhibit hysteresis: once formed, they persist and intensify even if the oscillations are removed.

Together, our results demonstrate that surface temperature oscillations have a strong impact on persistent mesoscale organization, especially in the realistic limit of fine spatial model resolution.

## 2 Materials and Methods

*Large‐Eddy Model and Boundary Conditions.* To simulate the convective atmosphere, we employ the *University of California, Los Angeles* (UCLA) Large‐Eddy Simulation (LES) solver with sub‐grid scale turbulence parameterized after Smagorinsky (1963). The Coriolis force and the mean wind are both set to zero. Radiation effects are incorporated using a delta four‐stream scheme (Pincus & Stevens, 2009) and a two‐moment cloud microphysics scheme (Stevens et al., 2005). Rain evaporation depends on ambient relative humidity and the mean and spread of hydrometeor radii (Seifert & Beheng, 2006). Radiation interacts with the atmosphere including clouds, but does not impact the surface temperature, which is prescribed and spatially homogeneous. The prescribed surface temperature *T*_*s*_(*t*) is spatially homogeneous but oscillates temporally as

(1) $$T_{s}\left(t\right)=\bar{T_{s}}-T_{a}\;\cos\left(2\pi \;t/t_{0}\right),$$

where $\bar{T_{s}}=298\;K$, *t*_0_ = 24 *h* is the period of the simulated model day, $\bar{T_{s}}$ is the temporal average and *T*_*a*_ the amplitude of *T*_*s*_(*t*). For the simulations “DIU” *T*_*a*_ = 5 *K* is chosen, whereas for “RCE” *T*_*a*_ = 0. Insolation *S*(*t*) is taken as spatially homogeneous for all simulations. For the simulations “DIU” the insolation cycle *S*(*t*) oscillates temporally with an amplitude typical for the equatorial continent. For the “RCE” simulations, both *T*_*s*_(*t*) and *S*(*t*) are set constant to their respective temporal averages, that is, $T_{s}\left(t\right)=\bar{T_{s}}=298\;K$ and $S\left(t\right)=\bar{S}=445\;W\;m^{-2}$.

*Surface Latent and Sensible Heat Fluxes.* Surface heat fluxes are computed interactively by standard bulk formulae and increase with the vertical temperature and humidity gradients as well as horizontal wind speed. Horizontal surface wind speed is approximated through Monin‐Obukhov similarity theory (Stull, 2012). Our simulations use a simple parameterization of a homogeneous, flat land surface, by assuming surface latent heat fluxes to be reduced to 70% of those obtained for a saturated (sea) surface. For the DIU experiments, mean surface latent and sensible heat fluxes are *LHF* ≈ 57 W/m^2^ and *SHF* ≈ 18 W/m^2^, respectively, yielding a Bowen ratio of *B* ≈ 0.30, realistic for forested land.

**Figure 1:**
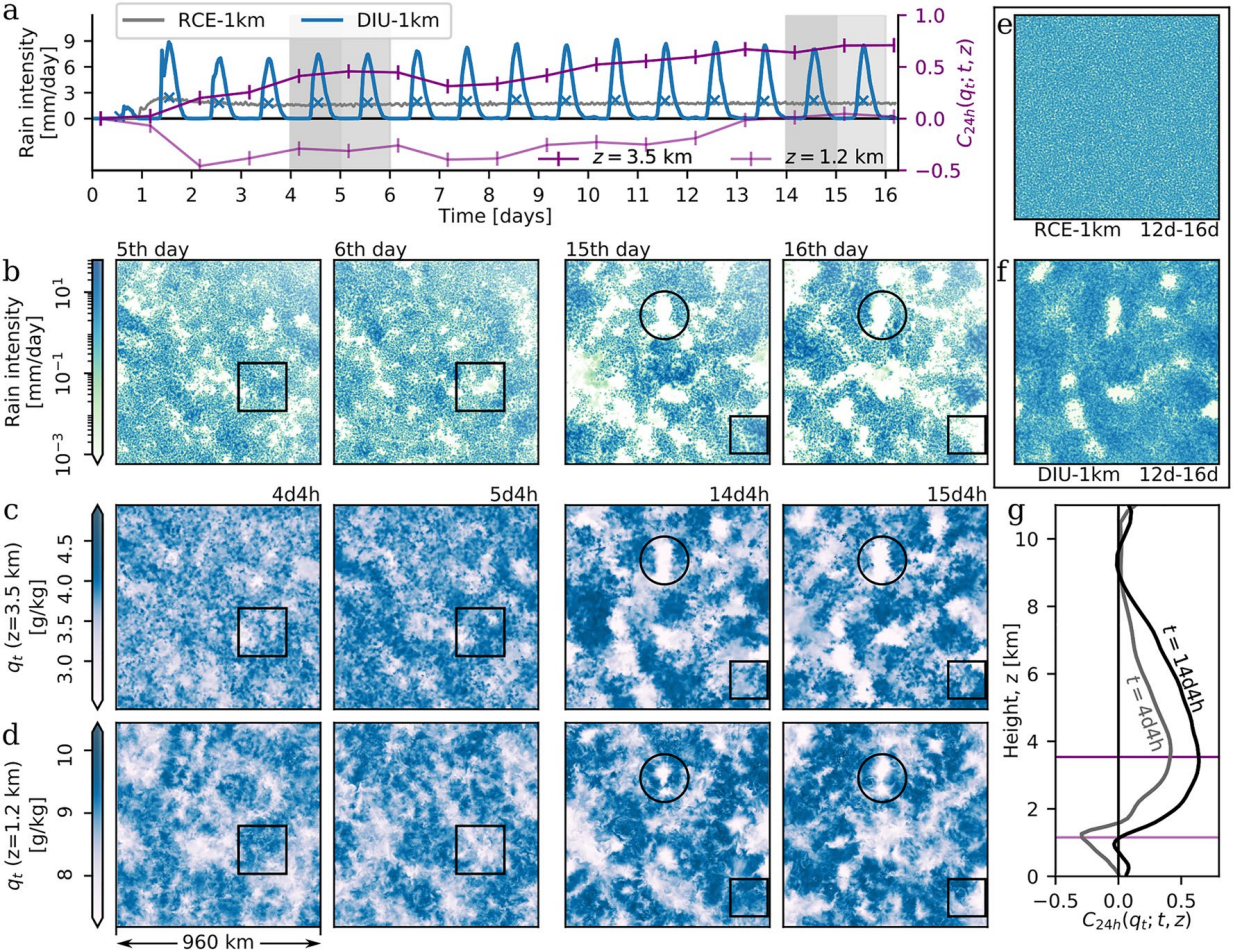
Spatio‐temporal organization by diurnal surface temperature oscillations. All data correspond to the DIU‐1 km experiment, except panel *e* and the gray curve in panel *a* which represent the radiative–convective equilibrium RCE‐1 km experiment for comparison. (a) Time series of domain‐mean rain intensity in DIU‐1 km (blue curve) and RCE‐1 km (gray curve). Blue ×‐symbols indicate daily‐average rain intensity in DIU‐1 km. Dark and faint purple curves show time series of 24‐hr Pearson correlations, *C*_24*h*_(*q*_*t*_; *t*, *z*), for total water mixing ratio, *q*_*t*_, for *z* = 3.5 km and *z* = 1.2 km, respectively, with *t* taken at 4 hr on any given day (*Details:* Methods). (b) Daily surface rainfall intensity for DIU‐1 km, temporally averaged over the 5th, 6th, 15th, and 16th day. Corresponding averaging periods indicated by gray shades in (a). (c) Early‐morning (4 hr) horizontal field of *q*_*t*_ (*t*, *x*, *y*, *z*) for *z* = 3.5 km for DIU‐1 km at times corresponding to the days in (b). (d) Analogous to (c) but for *z* = 1.2 km. Black squares and circles in (b)–(d) highlight regions discussed in the main text. (e) Four‐day average rain intensity on days 13–16 in RCE‐1 km. The color scale is the same as for panel (b). Note the lack of spatial organization. (f) Analogous to (e), but for DIU‐1 km. Note the rain‐free patches. (g) *C*_24*h*_(*q*_*t*_; *t*, *z*) vertical profiles, with *t* = 4d4h (gray) and *t* = 14d4h (black), respectively. The horizontal dark and faint purple lines indicate the respective vertical levels used in (a), (c), and (d). For animations of the entire time series corresponding to panels (a)–(d) *see* Movie.

*Initial Conditions.* Initial temperature and humidity are taken from observed profiles that represent potentially convective conditions (Moseley et al., 2016), but quickly self‐organize during the initial spin‐up. To enable the simulation to break complete translation symmetry, the initial temperature field is perturbed by small uncorrelated noise in the lowest model layer, drawn uniformly from the interval [−0.2, 0.2] K. The spin‐up manifests itself in “DIU” by relatively weak precipitation during the first model day, but relatively strong precipitation during the second. From the third day on, domain‐mean precipitation diurnal cycles are found fairly repetitive (*compare*: Figures 1a, S1, and Movies [Link], [Link], [Link], [Link], [Link], [Link], [Link], [Link]). Hence, over time, the system eventually establishes a self‐consistent vertical temperature and moisture profile.

*Model Grid, Dynamics, and Output.* The anelastic equations of motion are integrated on a regular horizontal domain with varying horizontal grid spacing *dx* and laterally periodic boundary conditions (Table 1). Vertically, the model resolution is stretched, with 100 m below 1 km, 200 m near 6 km, and 400 m in the upper layers. A sponge layer is implemented between 12.3 km and the model top, which is located at 16.5 km. Horizontal resolution *dx*, domain size, and output timestep Δ*t*_*out*_ vary (Table 1). At each output timestep, instantaneous surface precipitation intensity, as well as instantaneous horizontal fields of velocity and thermodynamic variables at various vertical levels are recorded. Three‐dimensional thermodynamic output data are recorded instantaneously at local solar time 4, 10, 16, and 22, whereas three‐dimensional velocities are recorded as time averages between local solar time 0–6, 6–12, 12–18, and 18–24. Additionally, at 30‐s and five‐minute intervals, respectively, spatially as well as horizontally averaged time series were extracted from the numerical experiments.

**Table 1:** Summary of Numerical Experiments

Experiment name	Surface temperature amplitude, *T*_*a*_ (K)	Hor. resolution *dx* (km)	Domain size *L* (km)	Simulation period (days)	Output timestep Δ*t*_*out*_ (min)
DIU‐500 m	5	0.5	480	0–10	15
RCE‐500 m	0	0.5	480	0–10	20
DIU2RCE‐500 m	0	0.5	480	9.75–17	15
RCE2DIU‐500 m	5	0.5	480	8.75–20	15
DIU‐500 m_small	5	0.5	240	0–18	15
RCE‐500 m_small	0	0.5	240	0–16	15
DIU‐1 km	5	1	960	0–16	20
DIU2RCE‐1 km	0	1	960	15.75–24	20
RCE‐1 km	0	1	960	0–16	20
DIU‐2 km	5	2	960	0–24	20
RCE‐2 km	0	2	960	0–20	20
DIU‐4 km	5	4	960	0–42	20
RCE‐4 km	0	4	960	0–20	20

*Note.* The term “DIU” is used to indicate simulations with diurnally oscillating surface temperature *T*_*s*_(*t*) and insolation *S*(*t*), whereas in “RCE” both *T*_*s*_(*t*) and *S*(*t*) are held constant. The term “DIU2RCE” means that “RCE” boundary conditions are applied as a continuation of “DIU” for the respective previous period—such as DIU2RCE‐500 m, which is initialized with the three‐dimensional atmospheric state after 9.75 days. The experiment names further include the respective horizontal model resolution.

*Temporal Correlation.* We define the 24‐hr lag correlation *C*_*24h*_(*q*_*t*_; *t*, *z*) used in Figures 1 and 7, S1, and S5 in Supporting Information S1 as the pixel‐by‐pixel Pearson correlation between time *t* and *t* + 24 hr, of the horizontal moisture distribution at the vertical level *z*:

(2) $$C_{24h}\left(q_{t};t,z\right)\equiv \sum_{i,j=1}^{N}\tilde{q_{t}}\left(t,x_{i},y_{j},z\right)\;\tilde{q_{t}}\left(t+24h,x_{i},y_{j},z\right)$$

where *N* = *L*/*dx* is the number of grid‐boxes along the domain side length (*see* Table 1). The relative spatial anomalies of $q_{t}$ at time t are defined as $\tilde{q_{t}}(t,x,y,z)\equiv \Delta q_{t}\left(t,x,y,z\right)/\sigma_{q_{t}}\left(t,z\right)$, where $\Delta q_{t}(t,x,y,z)\equiv q_{t}\left(t,x,y,z\right)-\langle q_{t}\rangle \left(t,z\right)$ is the absolute spatial anomaly of $q_{t}$ and $\langle q_{t}\rangle \left(t,z\right)$ its horizontal average at time *t* and vertical level *z*,

(3) $$\langle q_{t}\rangle (t,z)\equiv \frac{1}{N^{2}}\sum_{i=1}^{N}\sum_{j=1}^{N}q_{t}\left(t,x_{i},y_{j},z\right)$$

and $\sigma_{q_{t}}^{2}$ is the horizontal variance

(4) $$\sigma_{q_{t}}^{2}\equiv \frac{1}{N^{2}}\sum_{i=1}^{N}\sum_{j=1}^{N}{\left(q_{t}\left(t,x_{i},y_{j},z\right)-\langle q_{t}\rangle \left(t,z\right)\right)}^{2}.$$

Note that, by definition, *C*_*24h*_(*q*_*t*_; *t*, *z*) is bounded to lie between −1 and +1. We compare horizontal fields of *q*_*t*_ (*t*, *x*, *y*, *z*) for various values of height *z* at *t* chosen to represent 4h, hence early morning, of each given day. For the diurnal cycle simulations, at this time of day the atmosphere is generally stably stratified, convective activity is at a minimum and the moisture field is maximally smooth. This is an advantage because we are interested in the large scale structures, and not the precise locations of individual raincells, that typically measure only few kilometers in diameter.

*Coarse‐Graining Procedure.* Coarse‐grained rain intensity fields, termed $\bar{R}$, are used in Figure 3 and S4 in Supporting Information S1 to compute the relative standard deviation RSD$\left(\bar{R}\right)$, that is, the standard deviation divided by the mean, as well as the exceedance probability of daily precipitation intensity, respectively. $\bar{R}(k,l,m)$ is a three‐dimensional array where each element represent a space‐time cube of horizontal interval of length *s* and temporal interval of duration *τ*, that is, a cube of volume *s* × *s* × *τ*. Hence,

(5) $$\bar{R}(k,l,m)\equiv \int_{k\tau }^{\left(k+1\right)\tau }\;dt\int_{ls}^{\left(l+1\right)s}\;dx\int_{ms}^{\left(m+1\right)s}\;dy\;\;R\left(t,x,y\right),$$

where *R* is the model output instantaneous rainfall intensity (Table 1). We choose *s* = 32 km spatially. Temporally, Figure 3 uses *τ* = 48 hr and Figure S4 in Supporting Information S1 uses *τ* = 24 hr. The interval *s* = 32 km is a compromise between being significantly larger than typical individual deep convective rain events yet small compared to the system size. The interval *τ* = 48 hr in Figure 3 is chosen to emphasize persistent structures and discount the day‐to‐day anti‐correlated, high intensity mesoscale rain clusters. The interval *τ* = 24 hr in Figure S4 in Supporting Information S1 is chosen to capture the natural timescale of 1 day and make contact to usual extreme event statistics.

*Lagrangian Particle Tracking.* The particle tracking used in Figure 4 works in the following way: we distribute a set of tracers over the lowest horizontal level (*z* = 50 m) on the morning of the second day (1d6h). The set forms a square lattice with one tracer placed every 4 km. The particles are then transported over a 24 hr‐period using the horizontal velocity solution and a trapezoidal method with a 15‐min timestep. We choose to analyze the second simulation day because: (a) The horizontal morning moisture distribution is increasingly clustered from day‐to‐day. Thus earlier days are preferable for disentangling the dynamical effects of cold pools from the thermodynamic preconditioning. (b) On the first day the rainfall is extremely sparse due to the spin‐up from the initial condition. Still, repeating the analysis on later days gives comparable results. In the DIU‐experiments we can seed tracers in the early morning when there is close to zero convective activity following the nocturnal cooling. This allows us to accurately capture the diurnal motion from onset of convection until the end of the last cold pools. In the RCE‐experiments there are no such silent periods, so we have to pick arbitrary beginning and end times for the tracers. An animation of this process is presented in Movie S9.

## 3 Results

How does the diurnal cycle affect the organization of convective clouds? To investigate this, we begin by examining two cloud resolving simulations, both with 1 km horizontal resolution: DIU‐1 km and RCE‐1 km. In DIU‐1 km, the surface temperature oscillates sinusoidally with a 24hr‐period and a 5K‐amplitude to mimic the diurnal cycle (*Details*: Section 2). RCE‐1 km is a control experiment where the surface temperature is kept constant at the mean value of 298 K.

### 3.1 Two Layers of Convective Organization

After a short spin‐up period (∼48 hr), rain intensity remains nearly constant in RCE‐1 km (Figure 1a, gray curve and Movies S6c, and S6d). In DIU‐1 km, the oscillating *T*_*s*_(*t*) is reflected in oscillations in domain mean rain intensity, with a pronounced afternoon peak and mostly rain‐free nocturnal conditions (Figure 1a, blue curve, and Movie S2d , blue curve). Whereas RCE‐1 km shows no sign of CSA (Figure 1e), the spatial pattern emerging in DIU‐1 km is strikingly different:

1. During the first few days, the rainfall develops a patchy mesoscale pattern with rain clusters measuring on the order of one hundred kilometers across (Figure 1b, fifth and sixth day, *compare* Movie S2) in compliance with previous findings (Haerter et al., 2020).
2. However, 10 days later (days 15 and 16), the dynamics follows a more complex pattern: in addition to the now larger and more intense MCSs, the domain is also spotted with persistently rain‐free patches (marked as circled areas in Figures 1b–1d and visible in Movie S2).

This persistent lack of rainfall becomes particularly clear when considering rainfall averaged during multiple days (Figure 1f) or in animations (Movies S1 and S2). The spatio‐temporal pattern found in DIU‐1 km (Figures 1b–1d and Movies S2a–S2c) seems to be a composition of two motifs related to separate mechanisms:

1. First, a negative feedback inhibiting convective activity in areas where rain was particularly abundant the day before (examples are marked by squares).
2. Second, a positive feedback that can preserve inactivity from day‐to‐day (an example is marked by circles).

Visually, these two features become most apparent when observing the dynamics in Movies S2a–S2c, where dry anomalies (brown shades) are clearly alternating from day to day at the intermediate height level (Movie S2b). Concurrently, dry patches remain persistent at the higher level at *z* = 3.5 km (Movie S2a).

To analyze these two competing feedbacks further, we turn to the horizontal moisture field *q*_*t*_ (*z*, *t*) at each vertical model level *z* and describe its temporal evolution from night to night using the 24 hr‐lag correlation *C*_*24h*_(*q*_*t*_; *t*, *z*) (*Details:* Methods). We choose early morning (4 hr) as a reference because the moisture field is diffusively smoothed due to the absence of convective activity during the night. The vertical profile of *C*_*24h*_(*q*_*t*_; *t*, *z*) reveals an interesting dynamical structure of two pronounced extrema (Figure 1g): a global minimum near 1.2 km and a global maximum near 3.5 km, indicative of alternating moisture patterns at the lower but persistent moisture patterns at the upper level. In the course of the simulation, the correlations generally increase towards more positive values, and by day 14 even the minimum at 1.2 km takes positive values (Figure 1a).

The moisture pattern at the lower level—corresponding approximately both to the cloud base and the top of the boundary layer—closely mirrors that of the rainfall (*compare* Figures 1b and 1d and Movies S2b and S2c). Examples are highlighted by black squares: at 4d4h, there is a strong positive moisture anomaly at *z* = 1.2 km. On the following (fifth) day, that region receives intense rainfall, resulting in intense drying near the cloud base (at 5d4h). On the sixth day, the area receives almost no rainfall.

The positive 24 hr‐lag correlation in the free troposphere (*z* = 3.5 km) implies persistence from day to day. Indeed, inspecting an example of one persistently dry patch (circled in Figures 1b–1d), rainfall is absent in the same region during consecutive days (*compare*: Movie S2). Rather than replenishing the moisture within the persistently dry patches, rain clusters now appear to transport moisture elsewhere, undergoing day‐to‐day oscillatory dynamics that specifically avoids the dry patches. Hence, despite the initial day‐to‐day alternation in rainfall pattern, later days show sustained rain‐free sub‐regions—suggesting that the diurnal cycle opens a path to CSA. Indeed, the persistent dry patches closely resemble the early stages of CSA as observed in classical RCE studies. However, as demonstrated by our control experiment, RCE‐1 km, no signs of CSA occur in our numerical setup when the surface temperature is constant—at least not within 16 simulation days (*compare*: Movie S6). Therefore, we conclude that the diurnal cycle can trigger CSA under circumstances, where RCE cannot.

The dynamics described here is also observed in analogous simulations with horizontal resolution increased to 500 m. For this higher‐resolution case, the analog to Figure 1 is shown in Figure S1 (*compare*: Movie S1). There, dry patches form significantly more quickly and the negative peak of *C*_24*h*_(*q*_*t*_, *z* = 1,200 m) vanishes at an earlier time within the simulation. Again, such pattern formation cannot be seen in the constant‐temperature counterpart (*compare*: RCE‐500 m in Movie S5).

**Figure 2:**
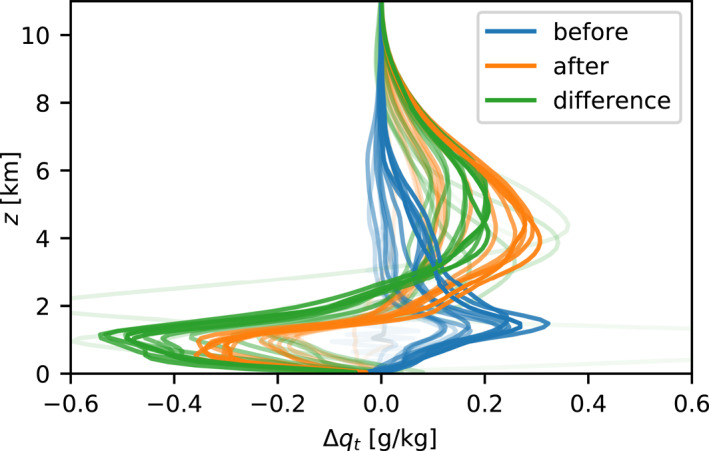
Moisture anomaly profiles before and after strong rain: On each of the first 15 simulation days in DIU‐1 km, the total daily surface precipitation is calculated for each column, and the top 10% are marked as “rainy.” Average moisture anomaly profiles for the “rainy” columns are then calculated at 4 hr the previous night (blue) and on the subsequent night (orange). The change in moisture from night to night, averaged over the “rainy” columns, is shown in green. Opacity increases linearly with time (faint curves represent early days).

The 24 hr‐lag correlation shows that different dynamics apply to moisture at different altitudes, but it does not reveal much about the physical processes. To explain the mechanism behind the correlations, it is useful to introduce the surface precipitation as an intermediary variable. Most vertical transport occurs in deep convective clouds, which leave a strong signature in surface precipitation. We therefore investigate the spatial correlation between rainfall on a given day and the moisture profiles on the preceding and subsequent nights (Figure 2). The blue curves show that strong rain is more likely to occur where the atmosphere was relatively moist in the early morning—especially around the cloud base (*z* ≈ 1.2 km) but also at higher levels of the free troposphere. The orange curves show that where it rains, the boundary layer (*z* ≤ 1.5 km) tends to dry out while the convective clouds tend to leave the free troposphere very moist. The green curves show that the average moisture tendency from night to night is indeed negative at low altitudes (*z* ≤ 2 km) but positive in the free troposphere (*z* ≥ 2 km) in the columns with top 10 percent daily rainfall.

Using surface precipitation—a proxy for convective activity—as an intermediary variable, we can explain the moisture 24 hr‐lag correlations as follows: high moisture at any altitude promotes convective activity. Convection lowers moisture in the boundary layer, giving rise to the negative correlations and the alternating DSA behavior. Convection also increases moisture in the free troposphere, which drives positive correlations and which can induce persistence.

### 3.2 Stronger Clustering at Higher Resolutions

It is well known that fine horizontal resolution can hamper or even prevent CSA in the classical RCE framework. To study how resolution affects the emergence of persistent dry patches in combination with the active diurnal cycle, we conduct a series of simulations of horizontal resolution varying between 0.5, 1, 2, and 4 km. For each case we contrast a setup where the surface temperature oscillates at an amplitude of 5 K (DIU) with a control experiment where the surface temperature is constant (RCE) (*Details:* Table 1).

CSA is characterized by large scale separation of the domain into dry areas without convective activity and moist areas with intense precipitation, but even patches of intense rainfall at scales of heavily‐populated areas, such as large urban areas, can threaten society. To quantify such spatial rainfall variability we compute the relative standard deviation (RSD) of a coarse‐grained rainfall field $\bar{R}$, where rainfall is block‐averaged over 32 km × 32 km horizontally and 48 hr temporally (*Details:* Methods). The 32 km spatial scale is practically relevant as it corresponds to the size of large metropolitan areas. RSD hence quantifies the degree to which rainfall fluctuates at this specific scale. The averaging procedure discounts both the small‐scale fluctuations related to individual raincells and the day‐to‐day alternation related to the DSA dynamics.

**Figure 3:**
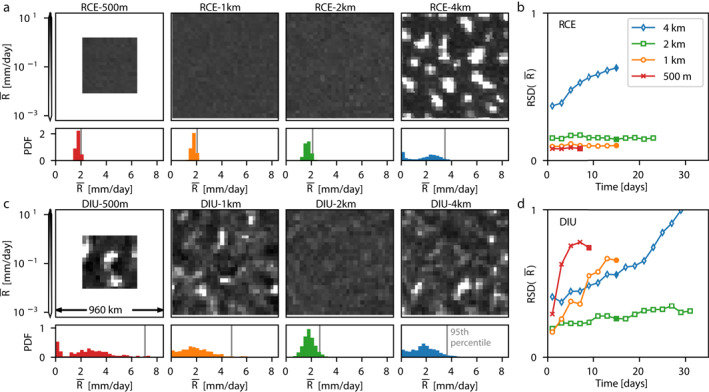
Faster diurnal aggregation at higher resolution. (a) Classical Convective self‐aggregation (CSA) under radiative–convective equilibrium (RCE) conditions, where the coarse‐grained rainfall field $\bar{R}$ is shown at decreasing resolutions. Note that spatial patterns only emerge at coarse resolution (4 km). The bar plots show the histogram of $\bar{R}$ for the corresponding field in each panel. In the histograms the gray vertical lines indicate the respective 95th percentile. (b) Time series of the relative standard deviation of $\bar{R}$ for each 48‐hr period for each of the RCE simulations in (a). (c, d) Analogous to (a) and (b), but for DIU. Note that strong phase separation, akin to CSA, now increasingly occurs at finer resolution. Time points used in (a) and (c): RCE‐500 m: *t* ∈ [6*d*, 8*d*], DIU‐500 m: *t* ∈ [8*d*, 10*d*], all other cases: *t* ∈ [14*d*0*h*, 16*d*0*h*], as indicated by the solid symbols in panels (b) and (d).

RCE‐4 km shows a clear trend of increasing RSD$\left(\bar{R}\right)$over time, a typical feature of classical CSA (Bretherton et al., 2005; Wing et al., 2017). However, for horizontal resolutions of 2 km or finer, the RCE experiments show no sign of CSA (Figures 3a and 3b) and RSD$\left(\bar{R}\right)$ remains constant over time. This result agrees with the existing literature, which generally states that in RCE simulations, CSA is inhibited by fine horizontal model resolutions (Muller & Bony, 2015; Wing et al., 2017).

In DIU, however, persistent rain‐free patches are visible at 1 km resolution, resulting in RSD$\left(\bar{R}\right)$ rapidly increasing over time. The increase is even more pronounced in DIU‐500 m, despite the smaller domain size which is also often considered detrimental to CSA (Jeevanjee & Romps, 2013; Muller & Held, 2012; Yanase et al., 2020) (Figures 3c and 3d). In DIU‐2 km, RSD$\left(\bar{R}\right)$ shows a weaker increase and persistent rain‐free patches are all but absent (Figures 3c and 3d). These observations lead us to conclude that the diurnal mechanism responsible for producing persistent dry patches is stronger with increasing horizontal resolutions. At our coarsest resolution, DIU‐4 km also aggregates at a rate similar to that of RCE‐4 km. However, based on the boundary‐layer dynamics (subsequent Section 3.3), we ascribe this to known CSA feedbacks, acting despite the diurnal cycle. Here we used the RSD of block‐averaged rainfall to quantify the degree of aggregation. Using *C*_*24h*_(*q*_*t*_; *z* = 3,500 m, *t*) as an alternative measure of persistent aggregation (Figure S5 in Supporting Information S1), shows that increased day‐to‐day persistence is commensurate with increases in RSD. The findings on RSD mean that the likelihood of experiencing extreme convective rainfall at the scale of large urban settlements increases strongly, when the diurnal cycle is pronounced and when model resolution is high (≤1 km horizontally).

The current study mainly focuses on the emergence of persistent dry patches, but it is worth noticing that the diurnal cycle has a significant impact in the opposite (high) end of the precipitation distribution as well. Extreme precipitation is strongly enhanced for high resolutions in DIU (*see* vertical gray lines in Figure 3c, lower panels, indicating the 95th percentiles). In RCE, extremes remain comparably small at such resolutions. The 99th percentile of daily rainfall—a typical index of extreme precipitation (Lenderink & Van Meijgaard, 2008)—increases fourfold in DIU compared to RCE at 500 m and 1 km horizontal resolutions, also measured at horizontal box‐size of 32 km × 32 km (Figure S4 in Supporting Information S1). Hence, precipitation extremes can be strongly impacted by diurnal surface temperature oscillations even though the daily average temperature is not changed.

### 3.3 Convective Cascades and Combined CPs

Why does convective activity aggregate at high resolution for DIU but not for RCE? This section describes the cascading dynamics leading to large MCS‐like rain events, combined CPs, and the day‐to‐day alternating spatial clustering described as “diurnal self‐aggregation” (DSA) (Haerter et al., 2020). Section 3.4 will follow up with a case study showing how the large scale fluctuations induced by the diurnal cycle can give rise to persistently dry patches, a pattern closely resembling the early stage of standard CSA.

**Figure 4:**
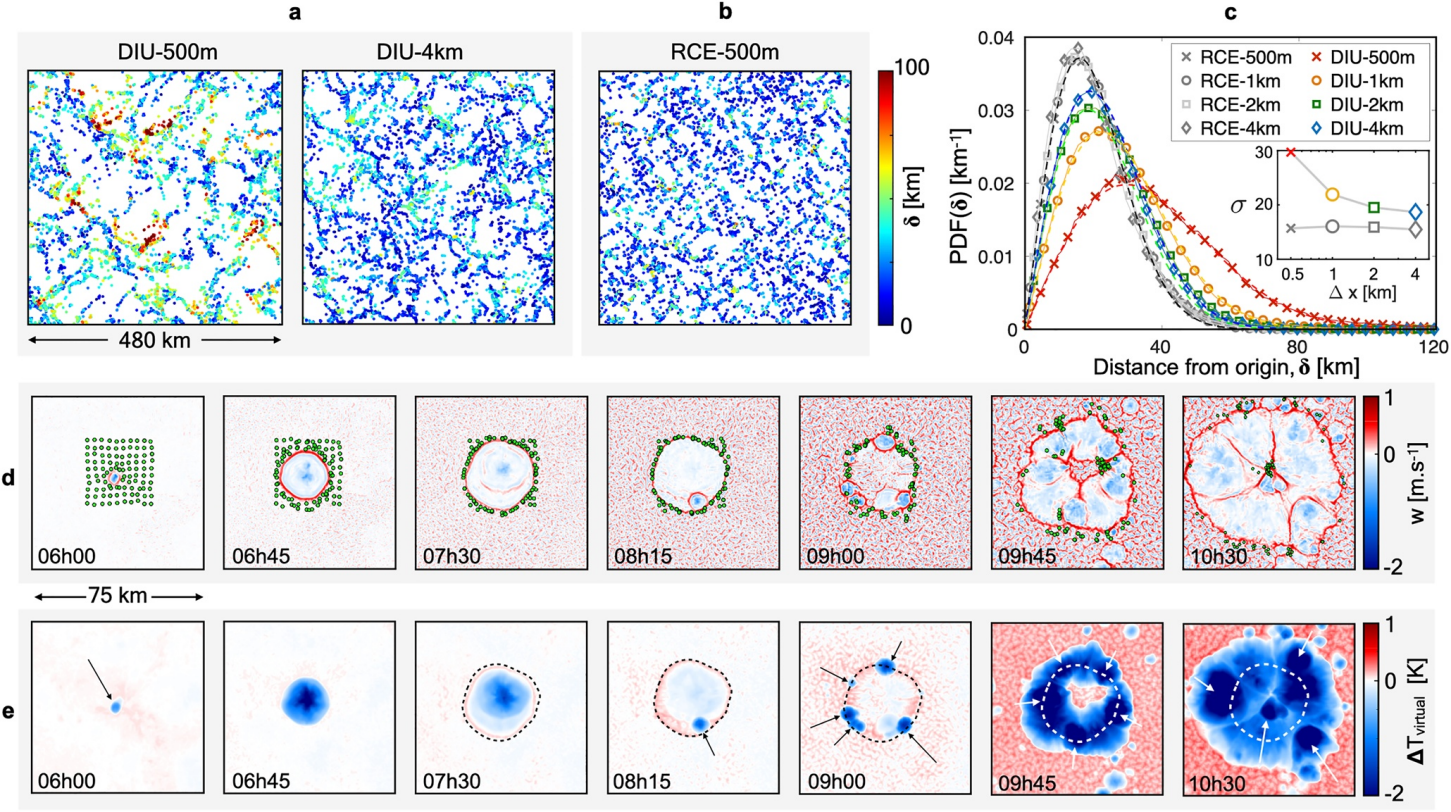
Low‐level circulation enhanced by resolution and diurnal cycle. (a) Final state of the Lagrangian particle tracking analysis in DIU. Tracers are seeded along a 4 km square lattice at 1d6h, and plotted at 2d6h and colored by the respective distance traveled, *δ*. (b) Analogous to (a) but using RCE‐500 m. All RCE cases yielded a visually similar particle field and are not presented for conciseness. (c) Probability distribution functions of *δ*. Dashed lines correspond to Rayleigh distribution best‐fits. *Inset:* Scale parameter *σ* versus horizontal resolution Δ*x* for DIU (colors) and radiative–convective equilibrium (RCE) (gray). The best‐fit scale parameters *σ* are (29.7, 21.9, 19.5, 18.7) km for DIU for *dx* = (0.5, 1.0, 2.0, 4.0) km, respectively, whereas *σ* = 15.7 ± 0.3 km for RCE. (d) Instantaneous vertical velocity fields at *z* = 50 m during day 1 (exact times as labeled) showing the evolution of a *primo*‐CP. 10 × 10 tracers, uniformly distributed on a square lattice with a spacing of 4 km, are initialized at 5 hr to visualize the surface flow. (e) Analogous to (d) but for the virtual temperature anomaly field Δ*T*_virtual_ defined as the local difference to the (*z* = 50 m)‐horizontal average. Arrows highlight new CPs. The dashed line, shown in several panels, corresponds to the convergence ring of the *primo*‐CP front once it has stopped its first expansion phase (*Details*: Methods).

We examine the cascade of events leading to different organizational patterns in DIU versus RCE by mapping the low‐level horizontal flow using Lagrangian particle tracking. Tracers are spaced regularly at time 1d6h, before the onset of precipitation, and passively advected with the horizontal flow during 24 hr (*Details*: Methods). In DIU‐500 m the final particle positions are visually clustered into stringy structures (Figure 4a) while such patterns are less pronounced in the coarser‐resolution simulation DIU‐4 km. Also, particles in DIU‐500 m are generally displaced much further than in DIU‐4 km, with large cleared spaces opening up in DIU‐500 m (*compare* panels in Figure 4a). The final particle positions in RCE‐500 m appear homogeneously distributed with generally low displacements, which is caused by an isotropic surface velocity field resulting in random‐like motion. This is further evidentiated by the animation provided in supplemental material (Movie S5).

To quantify these differences between DIU and RCE, we compute the distances between the initial and final positions of each particle, termed *δ*. The set of distances for all particles yields an empirical probability density function of *δ* (Figure 4c) for each simulation. These distributions are all well‐fitted by Rayleigh functions, *P*(*δ*) = *δ*/*σ* ^2^ exp (−*δ* ^2^/2*σ* ^2^) (shown in dashed lines), which is consistent with the motion of a random walker. The diffusive length scale *σ* measures the typical distance traveled. Interestingly, *σ* systematically increases for DIU as the model resolution is made finer (Figure 4c, inset). In RCE, not only do particles cluster less than in DIU (Figures 4a and 4b), but the resolution has no effect on *σ*: it consistently remains smaller than for DIU (Figure 4c, gray curves vs. colored curves). The continued increase of *σ* with finer resolution across all diurnal cases suggests the progressive activation of a convective organizing process as smaller length scales become explicitly resolved. Notably, the distribution of *δ* continues to broaden strongly, even between DIU‐1 km and DIU‐500 m, suggesting that numerical convergence has yet to be attained.

We now ask: can we identify a process which (a) improves low‐level circulation, (b) requires a diurnal cycle and (c) contributes to the organization of convection?

First, note that the RCE simulations are characterized by seemingly random ”eruptions” of convective raincells and the associated spread of CPs with typical diameters of ∼10 km. These CPs are the main cause of horizontal winds, and they quickly transport tracers from their interior to the gust fronts. However, the disorganized occurrences of new CPs prevent the tracers from traveling long distances effectively. This is evidenced by the small *σ* ∼ 15.7 km evaluated from all RCE (Figure 4c).

Second, a fundamental difference between RCE and DIU is the daily convection reset induced by nocturnal cooling. As a result, raincells in DIU occur in isolated diurnal bursts following the morning rise of surface temperature. One such early‐morning burst is now described step by step (see Figures 4d and 4e):

1. *6h00*: as a result of morning heating, a primordial raincell forms, typically in a location of moderately enhanced moisture near the top of the boundary layer. Having “won” the daily *race to precipitation*, this specific location produces a cold pool—the first one of this day’s precipitation cycle—which we term *primo‐CP.*
2. *6h45–7h30*: with no other CPs in the vicinity “vying” for the same space, the primo‐CP expands freely in a nearly‐undisturbed environment. Hence, the primo‐CP’s gust front propagates in a near‐circular fashion until it has exhausted all of its negatively‐buoyant potential energy. In this process it forms a structure we term *convergence ring*, marked by the green tracers and dashed lines in Figures 4d and 4e, respectively.
3. *8h15‐9h45*: by further destabilizing the surrounding environment through positive vertical velocity, the primo‐CP eventually sets off a cascade of secondary raincell–CP pairs along its convergence ring.
4. *10h30*: the secondary CPs instigate a tertiary population further away from its epicenter and so forth. Note that the primo‐CP is still fast expanding at this stage.

Such convective cascades transport passive tracers over large distances (see green tracers in Figures 4d and 4e), often exceeding 40 km (Figure 4c, red curve). Yet, as resolution is coarsened, the DIU *δ*‐distributions (Figure 4c) collapse back onto the RCE (hence cascade‐free) solutions. This is evidence that the cascade mechanism weakens for low resolution. This is not surprising, as it builds from the interactions of numerous smaller cold pool fronts, which are prone to numerical dissipation on coarse meshes (Grant & van den Heever, 2016).

Third, since convective cascades last for several hours and often span over ∼100 km horizontally, we view them as emergent MCSs (Houze Jr., 2004). This process is driven by outward‐running fronts merging into an enclosing macro‐structure, which we refer to as a *combined CP* (Haerter et al., 2020). The large areas of the combined CPs result in a more persistent tracer transport (green points in Figure 4d), explaining the broader *δ*‐distributions. Similar MCS‐like expansion processes occur throughout the model domain, on the same and on subsequent days. Eventually, later in the day, the interaction with other combined CPs and the decreasing surface heating halt further expansion. After the cascade is completed, a large region remains convectively suppressed by the reduced buoyancy of the cold and dry boundary layer (Figures 4d and 4e, 10h30): as such, combined CPs are responsible for the negative day‐to‐day correlations of rainfall.

**Figure 5:**
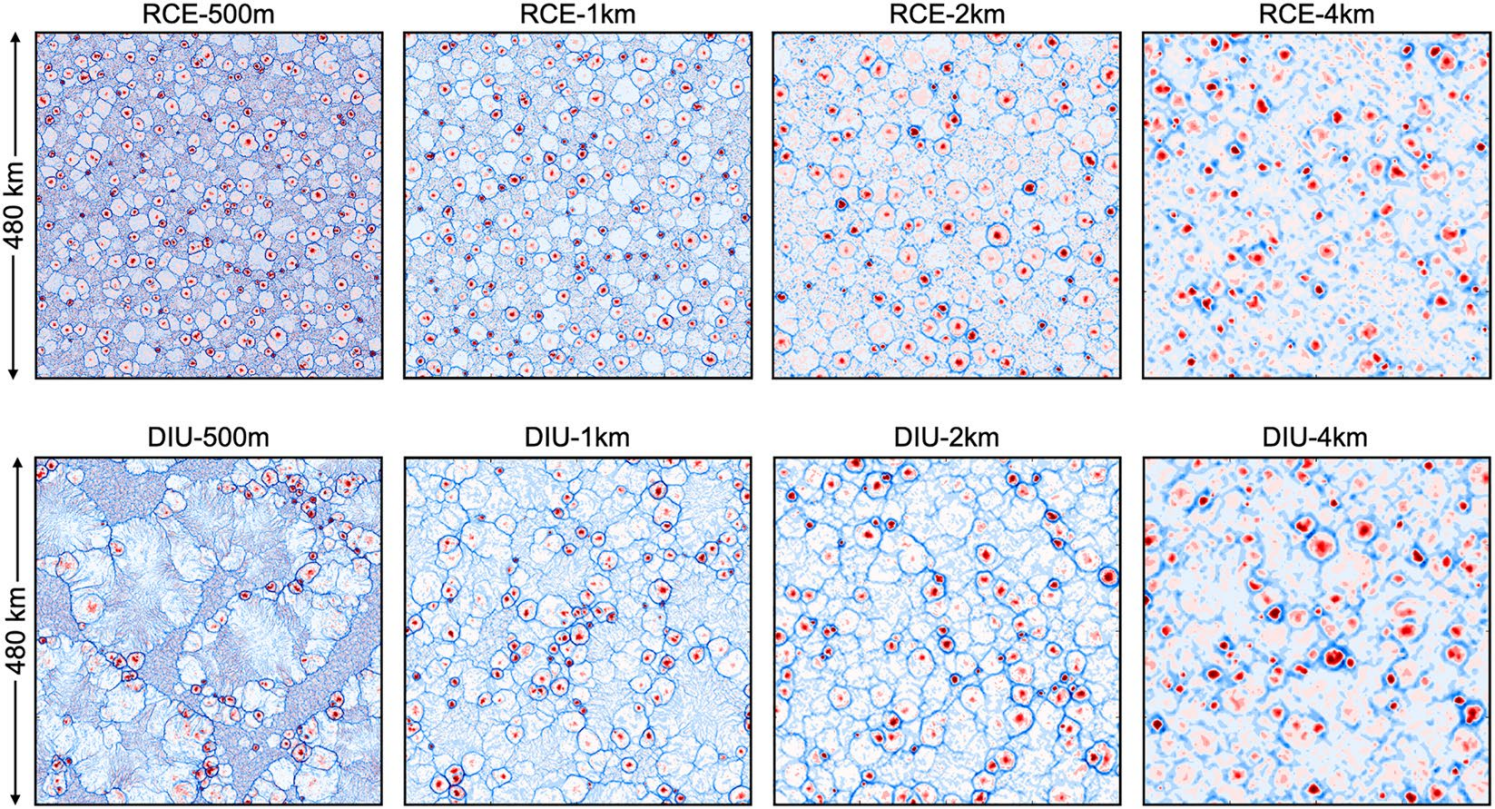
Contours of surface horizontal velocity divergence fields. Instantaneous horizontal fields of $\frac{\partial u}{\partial y}\;+\;\frac{\partial v}{\partial x}$ for the first model level (*z* = 50 m) at 18h00 on the sixth day. The contours range from −0.004 to 0.004 s^−1^ (blue to red) and are presented from left to right in decreasing order of spatial resolution for both (top) radiative–convective equilibrium and (bottom) diurnal configurations.

To conclude this analysis, we recall the two conditions to enable such cascades: (a) strong nocturnal cooling, ensuring quiescent conditions and a domain‐wide reset of convection —and thus the existence of a primo‐CP born from the first raincell in the morning. (b) a computational mesh sufficiently refined to resolve the convergence ring and the vertical mass fluxes at its edges—allowing the primo‐CP to transition into a combined CP. Note that (a) cannot be satisfied by RCE, thus hampering combined CPs. Conversely, (b) is likely not met by DIU‐2 km and DIU‐4 km where the coarse resolutions impede the cascading mechanism (*see* Figure 5 for illustration).

### 3.4 Genesis of a Persistent Dry Patch

**Figure 6:**
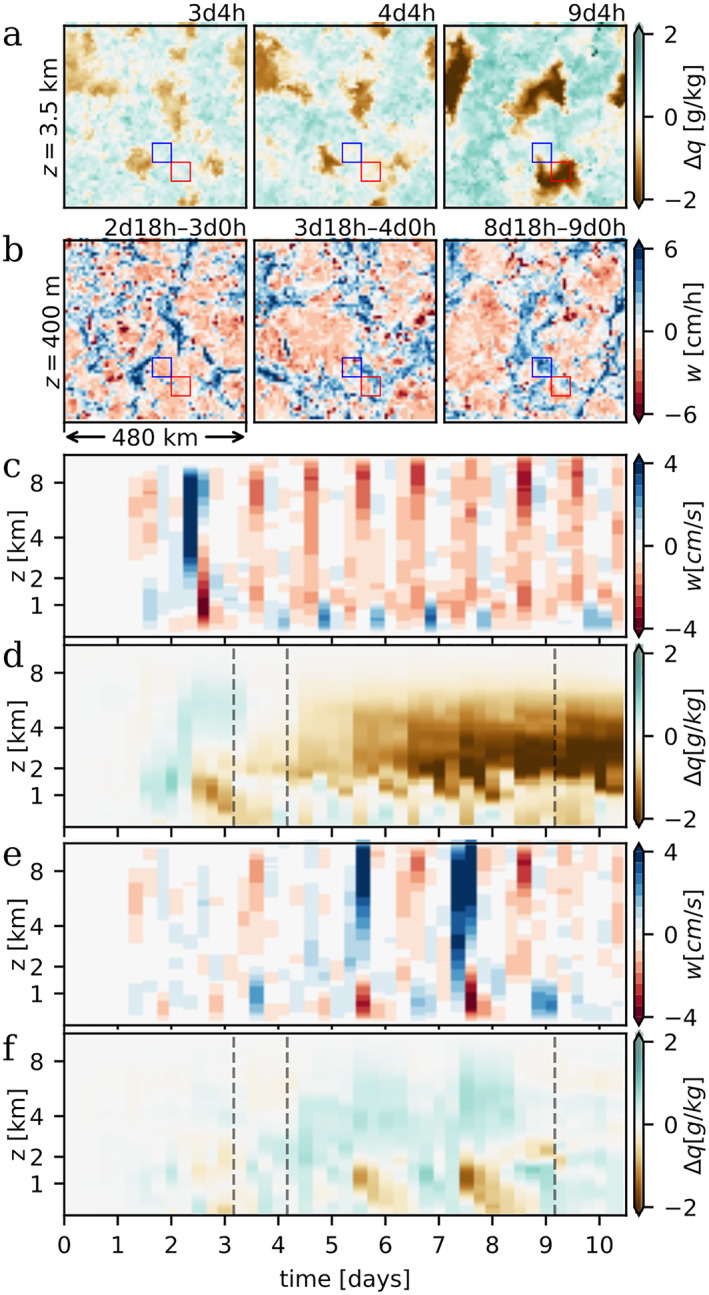
Onset of a persistent dry patch. All panels show data from DIU500 m. (a) Total water mixing ratio anomaly (Δ*q*_*t*_) at an altitude of *z* ≈ 3.5 km (free troposphere). The three snapshots are taken at 3d4h, 4d4h, and 9d4h, as marked by dashed lines in panels (d) and (f). A value of Δ*q*_*t*_ = 0 corresponds to the horizontal domain average at the given time and altitude. The red square marks a 50 km × 50 km region where a persistent dry patch emerges. The blue square marks a region which remains convectively active. (b) Vertical velocity (*w*) at an altitude of *z* = 400 m averaged in 6 hour intervals, 2d18h–3d0h, 3d18h–4d0h, and 8d18h–9d0h, and horizontally smoothed by a Gaussian filter with a scale parameter *σ* = 2 km. (c) Vertical velocity (*w* (*z*, *t*)) horizontally averaged over the area marked in (a and b) by red squares. (d) Total water mixing ratio anomaly (Δ*q*_*t*_ (*z*, *t*)) horizontally averaged over the same area as (c) (red squares). (e, f) Analogous to (c and d), but averaged over the area marked by blue squares.

The cascade process proposed in Section 3.3 describes how—at high resolutions—the diurnal cycle gives rise to spatially clustered rainfall patterns alternating from day to day. But how are dry patches enabled that persist even longer, over many days? In order to answer this, we will more closely consider the onset of one particular persistent dry patch in DIU‐500 m (Figure 6). The red squares in Figures 6a and 6b mark a 50 km × 50 km sub‐region where a dry patch emerges. By averaging variables horizontally within this region, we can visualize how the vertical structures and dynamics develop over time. The formation of the persistent dry patch is initiated by a strong updraft area at time *t* ≈ 2d12h (Figure 6c). This process leaves the free troposphere relatively moist, but the boundary layer is dried out (Figure 6d)—as is typical for columns with strong precipitation (Figure 2). On the following day, deep convection elsewhere forces pronounced subsidence within the region of interest. This subsidence leads to strong drying within the free troposphere, which experiences a change from a moist to a dry anomaly within a single day (Figure 6d, *t* ≈ 3d12h). At this stage, the resulting dry anomaly becomes self‐sustaining. Convective activity is inhibited by dryness (Figure 2) a feature attribute to the well‐known moisture‐radiation feedback invoked in studies on the maintenance of traditional CSA (Bretherton et al., 2005; Muller & Bony, 2015; Muller & Held, 2012; Yanase et al., 2020): the dry free troposphere (Figures 6a and 6d) gives rise to increased long‐wave cooling (*compare*: Figure S3i–S3l in Supporting Information S1), which in turn must be compensated by general subsidence heating. Subsidence further amplifies the drying and prevents deep convective activity. In the boundary layer, a circulation is driven by the CP outflow from surrounding deep convective activity (Figures 6b, 4d18h‐24h) and results in significant evening updrafts below *z* ≈ 1 km between days four and eight (Figure 6c). Such nocturnal low‐level updrafts, however, do not initiate new convection because the atmosphere is already stabilized at this time of day.

Outside the dry patches, the dynamics switches from day to day between two modes (Figures 6a and 6b blue boxes and Figures 6e and 6f): on some days, most noticeably the sixth and the eighth day, mid‐day convective activity results in heavy rainfall, increased free‐tropospheric moisture, and significant boundary layer drying. On other days, such as days 7 and 9, the dynamics resembles that within the persistent dry patch, with net mid‐day subsidence followed by low‐level nocturnal updrafts (Figure 6e). However, the mid‐day subsidence is weaker than within the dry patch, and the free troposphere does not become drier than the domain average. Therefore, convection is no longer inhibited once the boundary layer has re‐moistened. Rapid free‐tropospheric drying appears to be a pivotal step in persistent dry patch formation. By contrast, in RCE‐4 km and DIU‐4 km, the onset of persistent dry patches seems to be a more gradual process starting from small scale fluctuations (Figure S6 in Supporting Information S1).

### 3.5 Hysteresis of Aggregation (the Diurnal Trigger)

**Figure 7:**
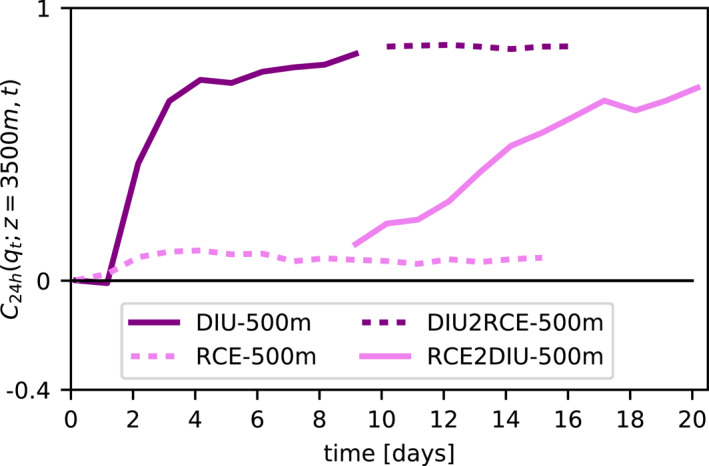
Hysteresis of CSA and the diurnal trigger. Time series of *C*_*24h*_(*q*_*t*_; *z*, *t*) (*Details:* Methods) at *z* = 3.5 km. Solid and dashed curves represent oscillating and constant surface temperature simulations. Dark purple curves represent a numerical experiment, where initially oscillating surface temperatures (solid) are set constant after 9.75 days (dashed). Lighter purple curves represent a numerical experiment, where initially constant surface temperatures (dashed) are replaced by oscillating surface temperatures after 8.75 days.

The numerical experiments discussed so far made use of spatially homogeneous initial conditions. One may wonder if structured initial conditions influence the emergence of persistent dry patches in DIU‐500 m. To test for the impact of initial conditions we run a new experiment, termed RCE2DIU‐500 m, where we use the state of the RCE‐500 m simulation at time 8d18h as an initial condition for a DIU simulation, where diurnal oscillations are then turned on. As Figure 6d suggests that persistent structures emerge from the free troposphere, we choose to quantify structure by the 24‐hr autocorrelation of moisture at *z* ≈ 3.5 km from night to night, *C*_*24h*_(*q*_*t*_, *z* = 3,500 km, *t*) (Figure 7). Once the oscillations are turned on, *C*_*24h*_(*q*_*t*_, *z* = 3,500 m, *t*) starts increasing almost immediately, suggesting that the oscillations are indeed an effective mechanism to form dry patches in the free troposphere. The dynamics in RCE2DIU‐500 m are qualitatively very similar to the dynamics in DIU‐500 m. Quantitatively, the positive correlation in the free troposphere increase somewhat more slowly and the negative correlations near the top of the boundary layer remain longer. We attribute these two differences to greater stability of the final state of RCE‐500 m compared to our uniform initial conditions.

By contrast, when continuing the RCE‐500 m simulation without diurnal oscillations, persistence remains small and constant for several additional days. Further, its final state is characterized by regular small‐scale moisture and rainfall patterns on the scale of individual cold pools, that is, approximately 10 km similar to that state shown in Figure 5. This finite simulation can of course not prove that a much longer simulation would never aggregate, but at least it demonstrates that the persistent structures form much faster when the surface temperature is oscillating. Hence, the diurnal cycle combined with a high‐resolution mesh activates a quick path to self‐aggregation.

Periodic surface temperature forcing can induce persistent dry patches, but can these dry patches prevail when the periodic forcing is removed? Such circumstances could come about in practice, when air masses are advected from land to sea, such as across the African west coast. To explore this, we now extend the DIU‐500 m simulation so that its state at time 9d18h serves as an initial condition for a simulation, termed DIU2RCE‐500 m, with the constant boundary conditions of RCE (Figure S2 in Supporting Information S1). The Pearson autocorrelation is nearly maximal for *q*_*t*_ (*z* = 3.5 km), already at the end of the 10‐day DIU‐500 m simulation. The switch to RCE gives rise to a persistently high autocorrelation. Additional analysis reveals that the classical CSA trademarks of increased long‐wave cooling and the absence of rainfall over dry areas (Bretherton et al., 2005) are all present for DIU2RCE‐500 m (Figure S3 in Supporting Information S1). Indeed, the state obtained after relaxation in DIU2RCE‐500 m shows that outgoing long‐wave radiation is increased over dry patches (Figure S3i–S3k in Supporting Information S1) and surface precipitation is all but absent (Figure S3n–S3p in Supporting Information S1). In summary, DIU2RCE‐500 m demonstrates that the atmosphere exhibits hysteresis: spatial patterns induced by a few days of diurnal oscillations can persist—and even intensify—without the oscillation. This result adds a new facet to a body of previous literature which also described hysteresis effects in relation to CSA (Holloway & Woolnough, 2016; Khairoutdinov & Emanuel, 2010; Muller & Bony, 2015; Muller & Held, 2012).

## 4 Discussion and Summary

This study set out to investigate the day‐to‐day alternating dynamics of MCS‐like precipitation clusters referred to as diurnal self‐aggregation (DSA) (Haerter et al., 2020). We found that the memory governing the negative precipitation autocorrelations can be traced back to the top of the atmospheric boundary layer, at *z* ≈ 1.2 km. However, the investigation also revealed a strong positive autocorrelation of moisture in the free troposphere, near *z* = 3.5 km. This possitive autocorrelation is associated with persistent dry patches closely resembling the onset of classical CSA. Such dry patches did not emerge in our control experiment with constant surface temperature and insolation. We therefore conclude that temporally oscillating surface temperature can give rise to persistent spatial symmetry breaking by triggering CSA.

*Diurnal Path Toward CSA—A Non‐Linear Instability.* The diurnal path to CSA is sensitive to horizontal model resolution. Performing simulations with varying horizontal resolutions (500 m, 1 km, 2 km, and 4 km), with and without surface temperature oscillations, our simulations showed that fine resolution favors CSA when the diurnal cycle is active. This is contrary to the usual constant surface temperature RCE framework where fine resolutions inhibit aggregation (Jeevanjee & Romps, 2013; Muller & Bony, 2015; Muller & Held, 2012).

We thus explored a time‐varying trend, visible at relatively short timescales of a few weeks. During this transient period, persistent symmetry breaking eventually occurred in some, but not all, simulations: In our diurnal cases persistent dry patches emerged faster at 500 m compared to 1 km resolution, and at 2 km resolution we did not observe persistent dry patches at all. At our coarsest model resolution (4 km), we found clear signs of classical CSA both in the main experiment (DIU) and in the control (RCE). However, the onset of CSA in DIU‐4 km more closely resembles the dynamics in the RCE‐4 km than the dynamics in the high‐resolution diurnal simulation. Interestingly, previous conceptual work attributed the onset of CSA to a linear instability in the free troposphere (Emanuel et al., 2014). By contrast, the onset of persistent dry patches in our high‐resolution simulations is a highly non‐linear process. It is initiated by strongly correlated cold pool dynamics in the boundary layer, a dynamics we term *convective cascades*. Such cascades form large combined cold pools which suppress convective activity for long enough to push the troposphere beyond the tipping point at which dry patches become self‐perpetuating.

*Strong Hysteresis.* The clustering dynamics revealed in the high‐resolution simulation was subject to strong hysteresis. In a separate experiment, DIU2RCE, we demonstrated that dry patches induced over a few days with a strong diurnal cycle can persist, and even intensify, once the surface boundary condition reverts to a constant surface temperature. This finding makes us speculate: when organized convective cloud clusters, produced under a high‐amplitude surface temperature forcing, are eventually advected over regions with little surface temperature variation, the clustered pattern may persist and even intensify further. Such a situation could be found at the interface between tropical continents and oceans, for example, at the west coast of Africa. One could imagine that spatial inhomogeneities are: induced by the strong continental diurnal cycle, advected out over the Atlantic Ocean by the easterly winds, and intensified despite the relatively weak sea surface temperature oscillations. However, more work is needed to clarify how large‐scale advection affects the convective organization in combination with the diurnal cycle.

*Summary.* To conclude, our numerical experiments showed that diurnal temperature oscillations can enable CSA under conditions that would not allow CSA given constant surface temperatures. This newfound diurnal path to CSA is even stronger in the realistic limit of fine model resolution and subject to strong hysteresis. Ultimately, we found that:

1. The memory governing the negative precipitation autocorrelations can be traced back to the top of the atmospheric boundary layer, at *z* ≈ 1.2 km.
2. The free tropospheric moisture field exhibits a strong positive autocorrelation associated with persistent dry patches, resembling the onset of classical CSA.
3. These persistent dry patches did not emerge in our mid‐to‐high‐resolution control experiments with constant surface temperature and insolation. Conversely, turning the diurnal cycle on in these control experiments activated the formation of dry patches.
4. Fine resolution favors CSA when the diurnal cycle is active.
5. The clustering dynamics revealed in the high‐resolution simulation is subject to strong hysteresis.

*Outlook.* We emphasize that these results rely on a set of highly‐idealized numerical simulations, where the surface temperature was prescribed. Our numerical setup should hence be seen as one of relative simplicity, where additional complexity resulting from a fully‐interactive land surface scheme, including soil moisture and vegetation feedbacks, was deliberately left out. Hence, we offer the following proposals how to further investigate diurnal cycle effects on convective organization:

1. *Larger Amplitude Oscillations.* Our simulations used a prescribed sinusoidal surface temperature oscillation with a diurnal range of 10 K around a mean of ${\bar{T}}_{s}=298$ K and a Bowen ratio of *B* ≈ 0.30 (*Details:* Section 2). These conditions were intended to mimic forested continental conditions in the deep tropics where the Coriolis force is weak enough to be neglected. Indeed, diurnal temperature ranges average approximately 10 K for tropical and subtropical evergreen forests. In non‐vegetated desert areas, however, daily fluctuations can be as large as 30 K (Sharifnezhadazizi et al., 2019). Future work should therefore explore how larger diurnal surface temperature amplitudes affect CSA, the formation of persistent dry patches and the spatio‐temporal extent of the emergent mesoscale convective systems. Such analysis may give additional insight into the formation and maintenance of mesoscale convective systems in more arid regions with strong diurnal surface temperature ranges, such as the Sahel (Parker & Diop‐Kane, 2017).
2. *The two‐way interaction* between a land surface and the local atmosphere should be explored using a radiative diurnal cycle, in a fashion similar to that in Hohenegger and Stevens (2016) and Tompkins and Semie (2020), albeit using sub‐kilometer horizontal resolutions and thinner slab layers, thus mimicking realistic continental diurnal cycles. In particular, the cooling effect of cold pools and cloud shading on the surface temperature should be incorporated within an interactive representation of the surface boundary condition. In RCE, recent studies have observed a negative feedback of surface responsiveness to CSA dynamics (Coppin & Bony, 2018; Shamekh et al., 2020). However, a highly‐responsive surface would also result in a larger diurnal temperature range, potentially overcoming the negative feedback. Such a study could also elucidate the formation of oceanic storms where the amplitude of diurnal heating measured by satellite may have been under‐estimated due to coarse resolution (Gentemann et al., 2008). It is therefore possible that diurnal sea surface temperature variations play a larger role than previously considered, especially in the extratropics where low‐wind conditions are most prevalent (Gentemann et al., 2008). Even near the equator, such as for the western Pacific warm pool, diurnal sea surface temperature variations of three kelvin have been documented during low‐wind conditions (Soloviev & Lukas, 1997). Given such evidence, temporal sea surface temperature variations might be sufficient to induce the persistent dry patches as we report them here.

*Closing Statement.* Convective organization has important consequences also beyond local precipitation extremes. In fact, numerical modeling of large‐scale thunderstorm clustering has been singled out as one of the fundamental questions relevant to the global climate (Bony et al., 2015). We hope that the current study will eventually help improve the connection between idealized experiments and empirical observations by raising awareness of the important effects resulting from temporal surface temperature fluctuations in general, and in particular the diurnal cycle.

## Supporting information

Supporting Information S1

Movie S1

Movie S2

Movie S3

Movie S4

Movie S5

Movie S6

Movie S7

Movie S8

Movie S9

## Data Availability

At http://doi.org/10.5281/zenodo.4898182 a subset of the data produced within this study is available. These data contain summary time series as well as time series of vertical profiles of a number of variables for all 500 m simulations.
