# Supplementary material for: The Diurnal Path to Persistent Convective Self‐Aggregation
Source: J Adv Model Earth Syst. 2022 May 23;14(5):e2021MS002923. doi: 10.1029/2021MS002923 (PMC9286477; doi:10.1029/2021MS002923)
Supplement: Supplementary file 1 — Supporting Information S1 [file JAME-14-0-s007.pdf]

Supporting Information for "The diurnal path to persistent convective self-aggregation"

Gorm G. Jensen[1], Romain Fiévet[1], Jan O. Haerter[1,2,3]

[1]Niels Bohr Institute, Copenhagen University, Blegdamsvej 17, 2100 Copenhagen, Denmark
[2]Complexity and Climate, Leibniz Center for Tropical Marine Research, Fahrenheitstrasse 6, 28359 Bremen, Germany
[3]Jacobs University Bremen, Campus Ring 1, 28759 Bremen, Germany

Contents of this file. This Supporting Information contains additional figures and a movie relevant for some specialist readers.

1. **Figures S1—S6**. Please refer to the individual figure captions for further details.
2. **Movies S1—S8: Animations for moisture and precipitation fields.** Files correspond to the moisture and precipitation fields for DIU-500m, DIU-1km, DIU-2km, DIU-4km, RCE-500m, RCE-1km, RCE-2km, and RCE-4km, respectively. In each animation the panels (a)-(c) show the following content: **a** Total water mixing ratio anomaly at 3500 m altitude (model level 30). **b**, Total water mixing ratio anomaly at 1250 m altitude (model level 12). **c**, Exponential moving average (EMA) of surface precipitation. A filter, defined by $(1-a)\sum_{t=0}^{T} a^{T-t} r_{int}(t)$, was applied to the timeseries. The constant a is chosen such that a 24-h old image contributes five percent relative to the current image: $a = 0.05^{1/B}$, where B is the number of timesteps per model day. **d**, Timeseries of domain mean surface precipitation. The red vertical line indicates the current time corresponding to the animations in (a)-(c).
3. **Movie S9: Lagrangian particle tracking.** Animation of the Lagrangian Particle Tracking analysis performed in Fig. 3 of the manuscript. Tracers are homogeneously distributed on the second day over the surface layer of three simulations (from left to right): DIU-500m, DIU-4km and RCE-500m. The tracers are transported using the horizontal surface velocity wind for a 24h-long period, and colored by their distance-from-origin. Note how 100km-long coherent circulation currents only form in the DIU-500m case.

Corresponding author: Jan O. Haerter, `haerter@nbi.ku.dk`

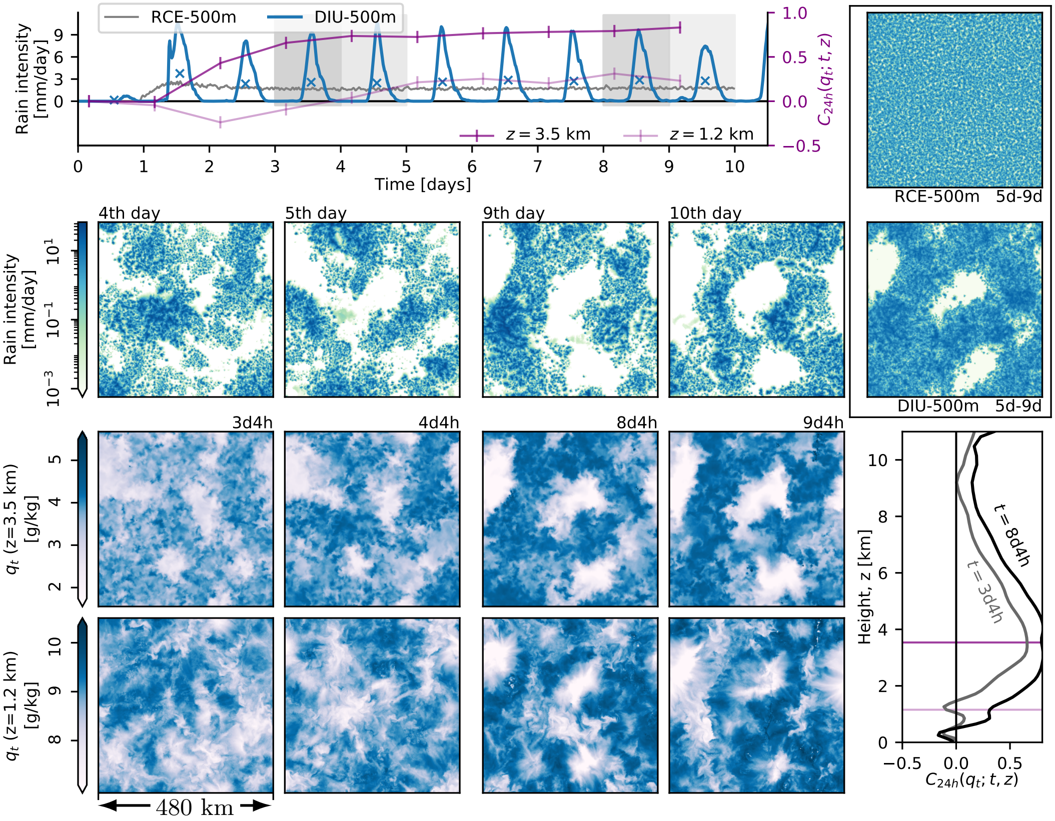

Figure S1. Spatio-temporal organization by diurnal surface temperature oscillations (DIU-500m). Analogous to Fig. 1, but for DIU-500m and RCE-500m.

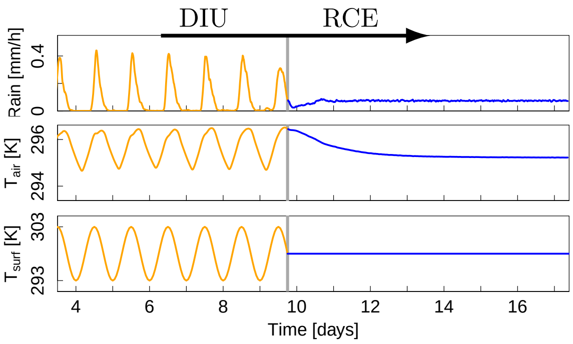

Figure S2. Domain mean time series for DIU2RCE-500m. Time series of horizontally-averaged rainfall intensity, near-surface air temperature ($T(z = 50\,m)$) and prescribed surface temperature (T_{surf}). Note the transition between temporally-varying (DIU) and temporally constant (RCE) surface temperature at t=9d18h, as well as the response in T_{air} and rainfall.

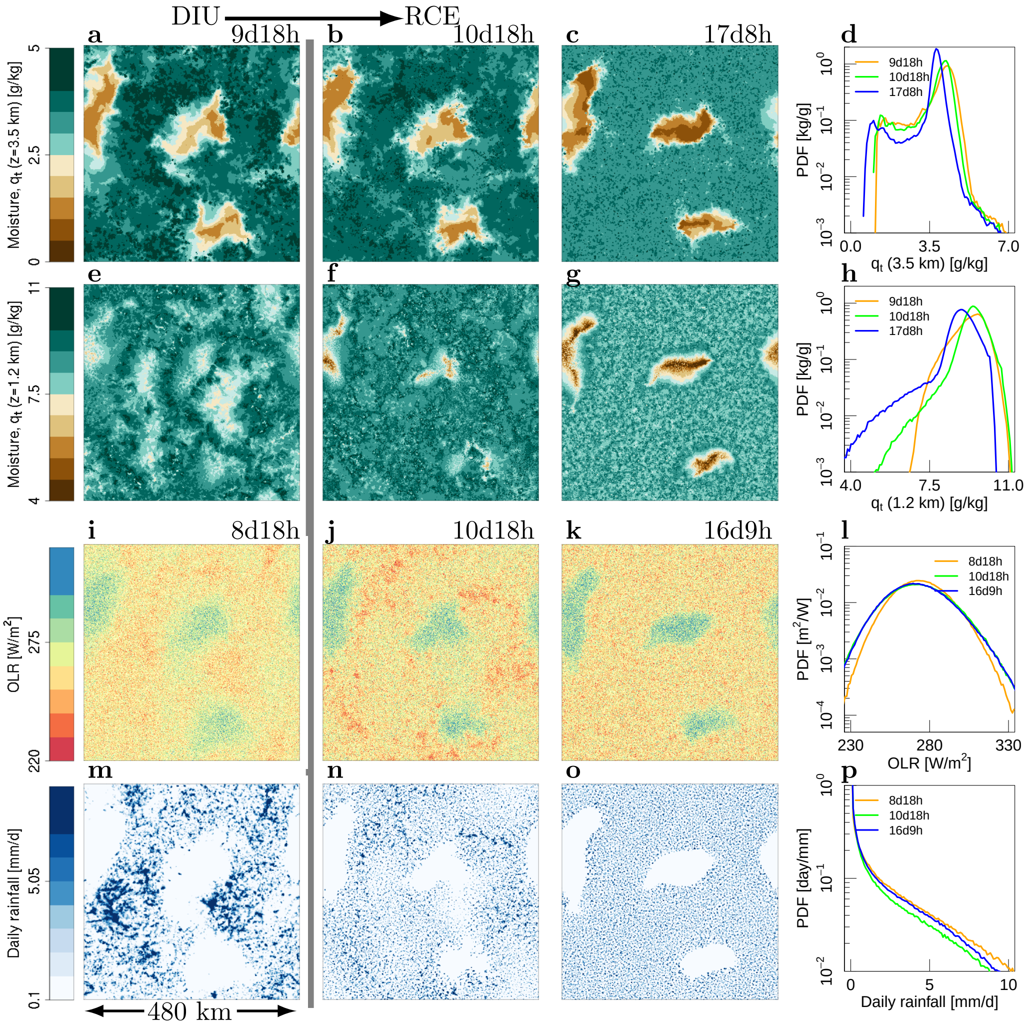

Figure S3. Transition from DIU to RCE. **a**, Water vapor mixing ratio anomaly $q'(z = 3.5\text{ km}) \equiv q(z = 3.5\text{ km}) - \overline{q_t}(z = 3.5\text{ km})$, where the overline denotes the horizontal mean, at the time of transition between DIU and RCE ($t = 9.75$ days) for DIU2RCE-500m. **b**, Analogous to (a), but one day after the transition to RCE ($t = 10.75$ days). **c**, Analogous to (a), but more than six days after the transition ($t = 17.35$ days). **d**, PDF of $q_t(z = 3.5\text{ km})$ for all three times shown in (a)–(c). Note the overall broadening and increasing bimodality of the humidity distribution function with time. **e**–**h**, analogous to (a)–(d), but for $q(z = 1.2\text{ km})$. **i**–**k**, Two-day temporal average (centered at the times noted on the panels) of outgoing long-wave radiation (OLR) at the model top. **d**, PDF of OLR, corresponding to the panels (i)–(k). Note the widening of the distribution function. **m**–**p**, Analogous to (i)–(l) but for rainfall intensity. Note the pronounced spatial structure in the rain field in (m) but the gradual relaxation to a featureless rainy sub-region and a rain-free dry region (n,o). In all histograms the vertical axis is logarithmic.

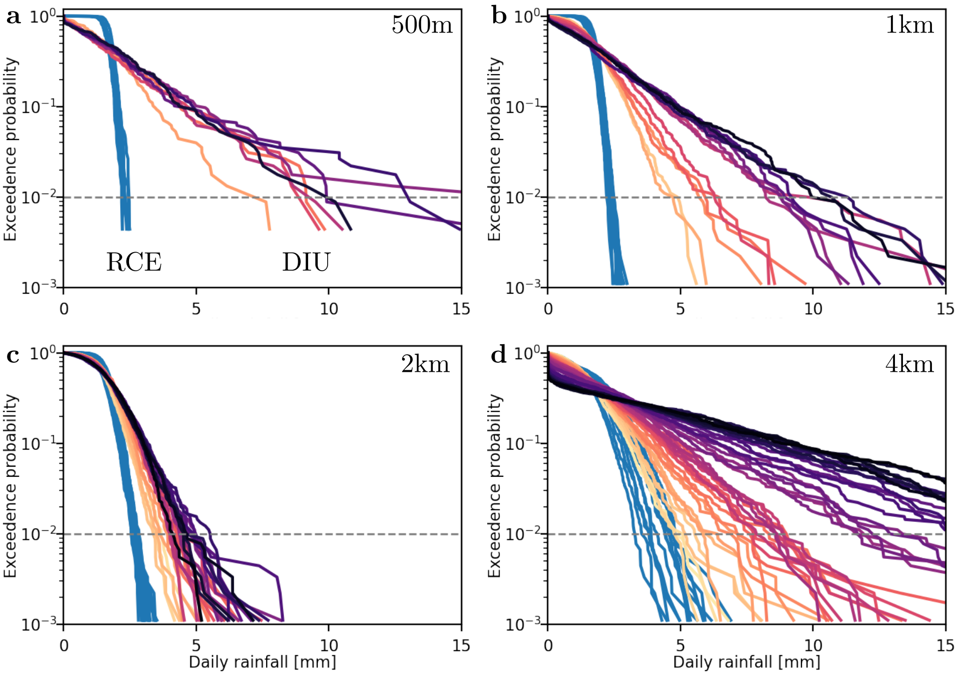

Figure S4. Daily accumulated precipitation distribution. The surface rainfall field R is coarse-grained to boxes of 32 km×32 km×24 hours (*Details*: Methods), representing daily rainfall averages with squares of 32 km side length. For various simulation day, we plot the normalized probability of such daily rainfall averages to lie above any given threshold. Curves of colors in the sequence of yellow-red-purple-black represent distribution functions for consecutive days. Analogously, the blue curves represent the RCE experiments. The first 48 hours are omitted in all datasets, to discard the spin-up period. Panels correspond to horizontal resolutions: **a**, 500m; **b**, 1 km; **c**, 2 km; **d**, 4 km. In all panels, the gray, dashed horizontal lines mark the 99th percentiles, as a typical measure of extreme precipitation. Note the pronounced increase in extreme precipitation for the later days of the DIU-simulation, where the 99th percentile reaches a multiple of that for the RCE-simulation. For all panels, note the logarithmic vertical axis scaling.

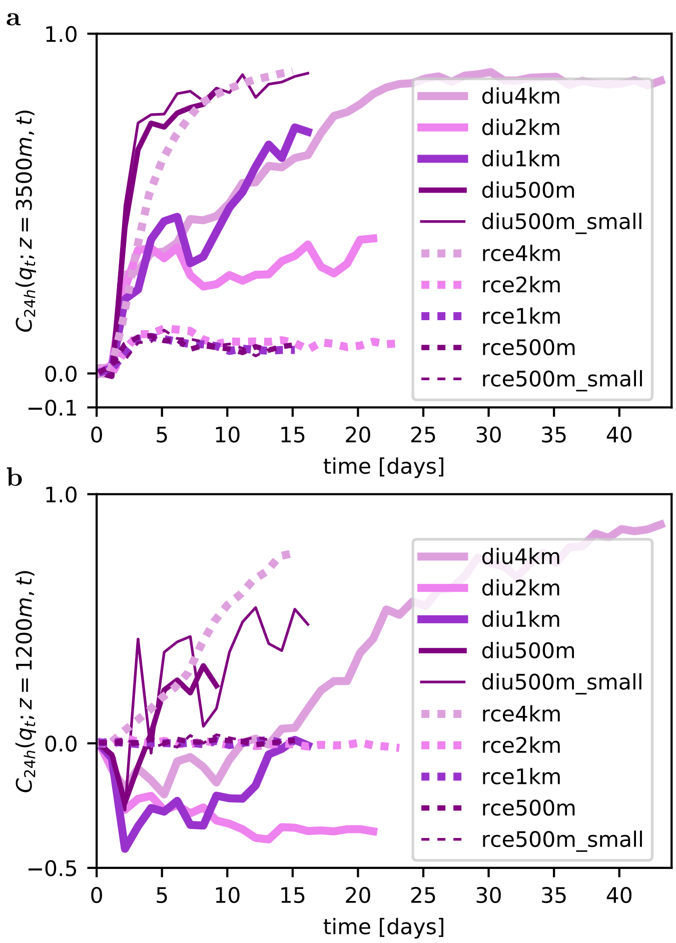

Figure S5. Persistent moisture anomalies in the free troposphere: Timeseries of $C_{24h}(q_t; z, t)$ at **a** $z \approx 3500$ m and **b** $z \approx 1200$ m for a series of simulations (*Details:* Methods). Dashed lines correspond to simulations in the RCE-setup (constant surface temperature and insolation), solid lines corresponds to DIU-experiments where the surface temperature oscillates with a 5 K amplitude and a 24 hours period. The different colors represent different horizontal model resolutions, and the line widths indicate different domain sizes (*Details:* Tab. 1). Strong positive correlations are associated with persistent dry patches and CSA. Notice that while the RCE setup only enables CSA at the coarsest model resolution (4 km), positive correlations build up very fast in DIU500m and DIU500m-small. In DIU-2km the correlation seems to stagnate around of $0.3 \sim 0.4$. In this simulation we also do not see persistent patches lasting for more than a few days at a time.

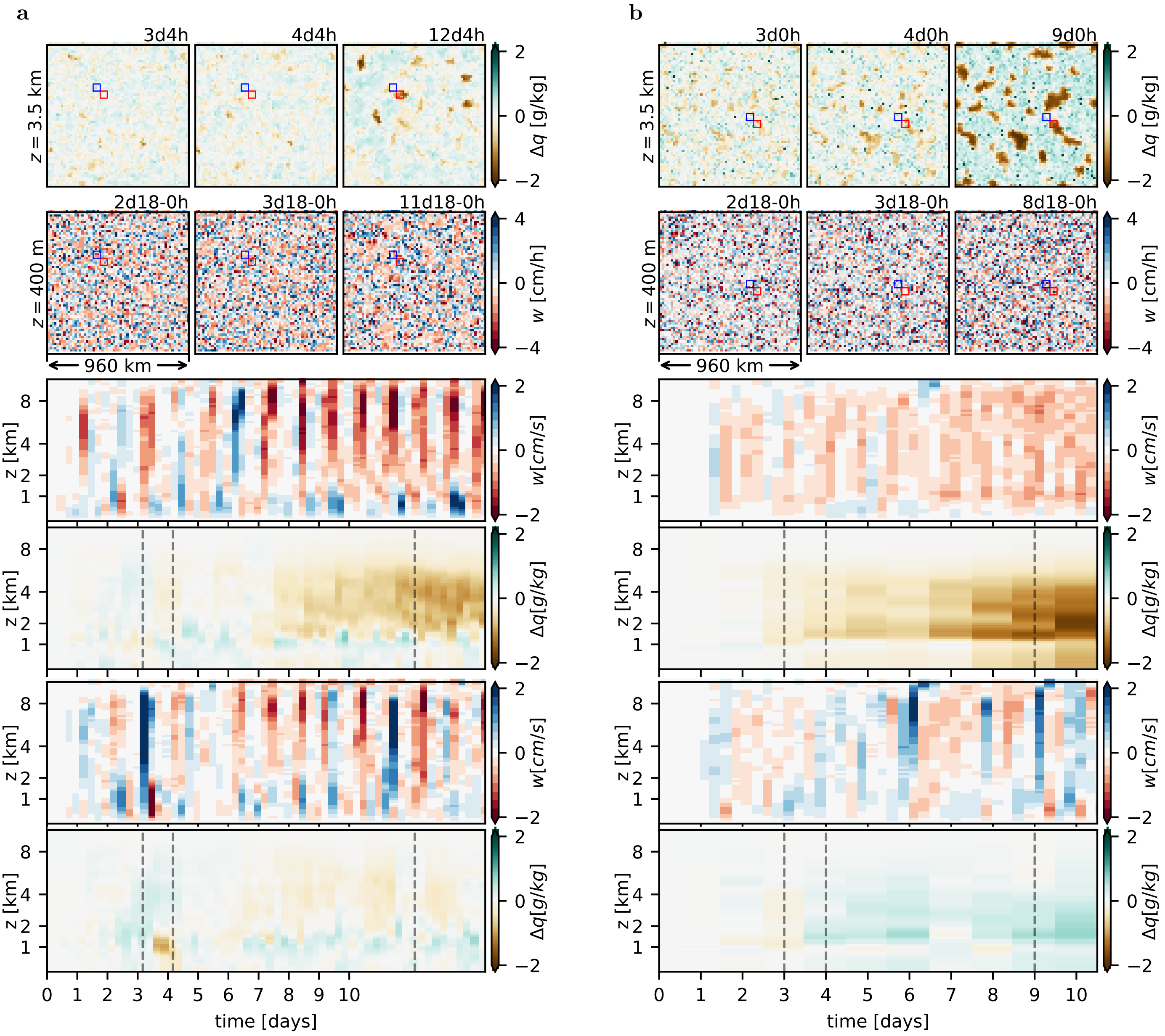

Figure S6. Onset of dry patches: Both figures are equivalent to Fig 6, but for two different simulations: **a** DIU-4km and **b** RCE-4km. The areas marked by red and blue squares are $48 \times 48\,\mathrm{km}^2$.
